# Defining publication bias: protocol for a systematic review of highly cited articles and proposal for a new framework

**DOI:** 10.1186/2046-4053-2-34

**Published:** 2013-05-21

**Authors:** Katharina Felicitas Müller, Matthias Briel, Alexandra D’Amario, Jos Kleijnen, Ana Marusic, Elizabeth Wager, Gerd Antes, Erik von Elm, Britta Lang, Edith Motschall, Viktoria Gloy, Guido Schwarzer, Doug Altman, Joerg J Meerpohl, Dirk Bassler

**Affiliations:** 1 Center for Pediatric Clinical Studies, University Children’s Hospital Tuebingen, Frondsbergstr. 23, 72070, Tuebingen, Germany; 2 Department of Neonatology, University Children’s Hospital Tuebingen, Calwer-Str. 7, 72076, Tuebingen, Germany; 3 Basel Institute for Clinical Epidemiology and Biostatistics, University Hospital Basel, Hebelstr. 10, CH- 4031, Basel, Switzerland; 4 School for Public Health and Primary Care (CAPHRI), Maastricht University, P.O. Box 6166200 MD, Maastricht, The Netherlands; 5 Department of Research in Biomedicine and Health, University of Split School of Medicine, Šoltanska 2, 21000 Split, OIB: 02879747067, Matični broj: 01315366, Žiro račun:2330003-1100071293, Split, Croatia; 6 SideView, 19 Station Road, Princes Risborough, Bucks HP27 9DE, United Kingdom; 7 German Cochrane Centre, Institute of Medical Biometry and Medical Informatics, University Medical Center Freiburg, Berliner Allee 29, 79110, Freiburg, Germany; 8 Cochrane Switzerland, IUMSP, University Hospital Lausanne, Biopôle 2, Route de la Corniche 10, CH-1010, Lausanne, Switzerland; 9 Institute of Medical Biometry and Medical Informatics, University Medical Center Freiburg, Stefan-Meier-Str. 26, 79104, Freiburg, Germany; 10 Centre for Statistics in Medicine, Botnar Research Centre, Windmill Road, Oxford OX3 7LD, United Kingdom

**Keywords:** Publication bias, Full publication, Underreporting, New framework, The OPEN project

## Abstract

**Background:**

Selective publication of studies, which is commonly called publication bias, is widely recognized. Over the years a new nomenclature for other types of bias related to non-publication or distortion related to the dissemination of research findings has been developed. However, several of these different biases are often still summarized by the term 'publication bias'.

**Methods/Design:**

As part of the OPEN Project (To Overcome failure to Publish nEgative fiNdings) we will conduct a systematic review with the following objectives:

- To systematically review highly cited articles that focus on non-publication of studies and to present the various definitions of biases related to the dissemination of research findings contained in the articles identified.

- To develop and discuss a new framework on nomenclature of various aspects of distortion in the dissemination process that leads to public availability of research findings in an international group of experts in the context of the OPEN Project.

We will systematically search Web of Knowledge for highly cited articles that provide a definition of biases related to the dissemination of research findings. A specifically designed data extraction form will be developed and pilot-tested. Working in teams of two, we will independently extract relevant information from each eligible article.

For the development of a new framework we will construct an initial table listing different levels and different hazards en route to making research findings public. An international group of experts will iteratively review the table and reflect on its content until no new insights emerge and consensus has been reached.

**Discussion:**

Results are expected to be publicly available in mid-2013. This systematic review together with the results of other systematic reviews of the OPEN project will serve as a basis for the development of future policies and guidelines regarding the assessment and prevention of publication bias.

## Background

Systematic reviews and meta-analyses of high-quality randomized controlled trials provide a valid summary of the available research findings, and are therefore crucial to evidence-based decision making [1]. It has long been recognized that the identification of the entire relevant research evidence is essential in order to come up with an unbiased and balanced summary. Thus, ideally all research activities conducted should be published and easily identifiable. As a second choice, the published studies should at least represent a random sample of all studies that have been conducted and the decision on publication should not depend on the nature and direction of the results. Only under such circumstances can systematic reviews and meta-analyses live up to their promise of providing unbiased, high-quality evidence for medical decision making. However, it is not always possible to retrieve all eligible evidence for a given topic, as many studies never get published. The phenomenon of non-publication of studies based on the nature and direction of the results has historically been referred to as 'publication bias' [2,3].

The public perception cannot only be distorted by the non-publication of an entire study, information may also be partially lacking or presented in a way that influences the take-up of the findings, such as selective reporting of outcomes or subgroups or 'data massaging' (for example, the selective exclusion of patients from the analysis). Thus, over recent years a new nomenclature for other types of bias related to the non-publication or distortion in the dissemination process of research findings has been developed, such as 'outcome reporting bias' [4], 'time lag bias' [5], 'location bias' [6,7], and many more. Nevertheless, all these different aspects are often still referred to as 'publication bias', although up to now no consensus on the definition of publication bias has been reached in the literature. Therefore, in this systematic review, we aim to summarize what is commonly understood by the term 'publication bias' and to propose a new framework on nomenclature of the various aspects of distortion in the dissemination process of research findings.

### Objectives

In terms of the above mentioned controversies regarding the definition of 'publication bias', we will conduct a systematic review with the following objectives:

• To systematically review highly cited articles that focus on non-publication of studies and to present the various definitions of biases related to the dissemination of research findings contained in the articles identified.

• To develop and discuss a new framework on nomenclature of various aspects of distortion in the dissemination process that leads to public availability of research findings in an international group of experts in the context of the OPEN Project (To Overcome failure to Publish nEgative fiNdings).

This systematic review will be part of the OPEN Project which was developed with the goal of elucidating the scope of non-publication of studies through a series of systematic reviews (for example, see [8]).

## Methods

### Systematic literature search

#### Search strategy

We will search Web of Knowledge [9] on a given day. We will use the simple search term 'publication bias'. We chose Web of Knowledge because it always follows a ranking according to the total number of citations and therefore allows us to identify the most frequently cited articles. Although we are interested in various aspects of bias in the dissemination process of research findings, our main aim is the identification of different definitions of publication bias and we thus decided that the term 'publication bias' should be part of all publications of interest. No language restrictions will be applied. We will not search any other database or any grey literature since our focus is on highly cited and publicly available articles in order to capture the most used definitions of publication bias.

#### Eligibility criteria

We will include the 50 most frequently cited articles that focus on biases related to the non-publication or distortion in the dissemination process of research findings from any source and addressed to any audience. We chose the number 50 arbitrarily and will not exclude self-citations because we are interested in the absolute number, independent of the people that cited the work. In order to be included, articles must include the term ‘publication bias’ and provide some form of definition of this phenomenon. We will only include fully published articles.

#### Study selection

Two reviewers will independently and in duplicate screen titles and abstracts of search results. If a title or abstract cannot be rejected with certainty by both reviewers, the full text of the paper will be retrieved and assessed for eligibility. Any disagreement among the reviewers will be resolved by discussion and consensus or, if needed, by third-party arbitration.

#### Data extraction

A specifically designed information extraction form will be developed and pilot-tested. Two reviewers will independently extract all relevant information from each eligible article. The following information will be collected:

o Baseline data (for example, author names, language and year of publication, journal)

o Number of citations in Web of Knowledge and rank

o Definitions of biases related to the dissemination of research findings

o Methods to detect, quantify and adjust for publication bias

o Suggestions to minimize publication bias

o Impact of publication bias

Any disagreement will be resolved by discussion and consensus or, if needed, arbitration by a third reviewer.

#### Data analysis and reporting

Data synthesis will involve a descriptive summary of the range of definitions given to describe various forms of biases related to the dissemination of research findings. We will report the study according to PRISMA (Preferred Reporting Items for Systematic reviews and Meta-Analyses) guidelines [10].

### Framework development

We will propose a draft capturing the ideas and experiences of the core group of authors. This draft will be complemented by our findings from the 50 most frequently cited articles in Web of Knowledge. We will then circulate a draft to an international group of experts that are part of the OPEN consortium. Each expert will review the draft and provide feedback regarding the issues we identified or contribute with other insights. We will continue this process until no additional ideas emerge.

At the end of this process we aim for consensus regarding the range of mechanisms that can distort the dissemination of research findings, its nomenclature and related definitions. We endeavor to provide a framework which focuses on the responsibilities of all players on the various stages of the dissemination process.

## Discussion

The Helsinki Declaration states clearly that 'Authors, editors and publishers all have ethical obligations with regard to the publication of the results of research. Authors have a duty to make publicly available the results of their research on human subjects and are accountable for the completeness and accuracy of their reports. […]. Negative and inconclusive as well as positive results should be published or otherwise made publicly available' [11]. However, many research results never get published. The non-publication of study results is of great importance because it distorts the evidence base for clinical decision-making, which is increasingly based on the synthesis of published research and might therefore lead to patients receiving an ineffective or even harmful treatment [12-15].

This systematic review seeks to give a broad overview of the various definitions currently used to describe biases related to the dissemination of research findings. Based on our findings, together with reflections from an international group of experts (the OPEN consortium), we aim to propose a new framework on nomenclature of various aspects of distortion in the dissemination process of research findings which focuses on the responsibilities of all players. We hope that this player-based framework will help to clarify responsibilities for publication and related bias.

Being part of the OPEN Project this systematic review, together with the results of other systematic reviews of this subject, aims to raise awareness of the significance of bias related to the non-publication or distortion in the publication process of research findings and the complexity of this issue. In addition, these reviews will also provide a foundation for a recommendations workshop, which will enable key members of the biomedical research community (for example, funders, research ethics committees, journal editors, etc.) to develop future policies and guidelines to minimize non-publication and related biases.

## Abbreviations

OPEN: To Overcome failure to Publish nEgative fiNdings; PRISMA: Preferred Reporting Items for Systematic reviews and Meta-Analyses.

## Competing interests

We declare that all authors and contributing members have no competing interests.

## Authors’ contributions

DB and JJM conceived of the study. DB and KFM designed the search strategies. KFM drafted the manuscript with the help of DB. KFM, MB, AD, JK, AM, EW, GA, EvE, BL, EM, VG, GS, DA, JJM, and DB critically reviewed the manuscript for important intellectual content. KFM, MB, JK, AM, EW, EvE, DA, JJM and DB will develop the new framework. All authors read and approved the final version before submission. KFM and DB are guarantors.
